# Oligometastatic Disease at Presentation or Recurrence for Nonsmall Cell Lung Cancer

**DOI:** 10.1155/2012/396592

**Published:** 2012-07-30

**Authors:** Daniel R. Gomez, Yuzuru Niibe, Joe Y. Chang

**Affiliations:** ^1^Department of Radiation Oncology, The University of Texas MD Anderson Cancer Center, 1515 Holcombe Boulevard, Houston, Unit 0097, TX 77030, USA; ^2^Department of Radiology and Radiation Oncology, Kitasato University School of Medicine, Sagamihara 252-0374, Japan

## Abstract

Oligometastatic Non-Small Cell Lung Cancer (NSCLC) presents a unique opportunity for potential curative therapy. Improved cancer staging using PET/CT, MRI, and future cellular and molecular staging with circulating tumor cells and/or molecular markers will identify more patients with truly oligometastasis disease that will benefit from definitive local treatment. Recent development of noninvasive local ablative therapy such as stereotactic radiotherapy makes it possible to eradicate multiple local diseases with minimal side effect. Novel systemic therapy may also control systemic spread and therefore make it possible to improve survival by eliminating local diseases. More research, particularly prospective studies, is ideally randomized studies are needed to validate the concept of oligometastasis.

## 1. Introduction

Oligometastatic (OM) disease refers to a limited metastatic burden [1]. The precise definition of this entity has varied among studies, but the clinical significance is that this subgroup of patients may represent a population in which definitive treatment is feasible. As a result, numerous studies have been performed over the past several decades attempting to identify patients with OM malignancies that have indolent disease, the optimal treatment strategies in this setting, and prognostic factors for long-term survival with aggressive local therapy. In this paper, we discuss the current data on the pathophysiology of OM non-small cell lung cancer (NSCLC), compare the prognosis of OM at diagnosis (synchronous OM disease) and at recurrence (metachronous OM disease), and provide a literature review of studies assessing the role of aggressive therapy in this context. Our goal is to provide the reader with an understanding of the spectrum of OM NSCLC and to provide information that will assist the practicing oncologist in selecting patients for combined systemic and local treatments versus palliative approaches alone.

## 2. Proposed Pathophysiologic Mechanisms of Oligometastatic Disease

Several investigators have attempted to elucidate the biologic mechanism of OM disease. These studies have previously been summarized well in two reviews by Hellman and Weichselbaum [2, 3]. In these reviews, the authors describe the multiple steps of metastasis, as influenced by factors such as the microenvironment and tumor diversity and as outlined specifically by Gupta and Massagué [4]. These steps are as follows (1) aggressive phenotype, (2) prerequisites such as invasiveness, (3) a favorable microenvironment due to factors such as angiogenesis and inflammation, (4) intravasation, (5) increased life in transit due to improved vascular adhesion and platelet association, (6) a favorable distant environment, (7) homing in on the metastatic target, (8) extravasation by motility and vascular remodeling, (9) survival in the distant site, and (10) cancerization of the stroma and colonization in the distant site. 

Given these steps in the development of metastatic disease, it follows that in an individual patient (microenvironment) and tumor, the capacity and timeframe to achieve individual steps may vary by histology, organ system, or concurrent intervention. For example, lung cancer is predisposed to metastasize to the brain, lungs, adrenal glands, bone, and liver, while a metastasis to a structure such as the bladder, pancreas, or colon is rare. This predisposition is dependent on both the genomic nature of cancer, the seed, and the microenvironment (capacity for vascular adhesion, level of hypoxia), the soil, at that site. 

In an illustrative example, Yachida et al. performed a multi-institutional study in which rapid autopsies were obtained of seven patients with terminal pancreatic cancer. All patients had metastatic deposits in at least two metastatic sites. The authors then compared the mutation status of the lesions in the metastatic sites with that of the index lesion. It was found that there were two types of mutations: “founder” mutations which were present in all samples from a given patient and “progressor” mutations present in one or more of the metastases but not in the index lesion. From this information, the authors were able to construct evolutionary maps of each patient's malignancy. Furthermore, the authors found that metastases at a given location had similar mutation signatures, and that the subclones could be placed in an “ordered hierarchy establishing an evolutionary path for tumour progression” [5]. Thus, extrapolating from pancreatic cancer, it appears as if the primary tumor is a mixture of geographically distinct subclones, and one could then infer that the presence of specific subclones dictates the extent, location, and timing of metastases. These findings set a basis for OM as a distinct entity of metastatic disease, with individualized treatment paradigms.

## 3. Synchronous versus Metachronous Oligometastatic Disease

Synchronous and metachronous OM represent two subsets of this disease. Particularly in the case of intrathoracic metastases, a dilemma for the treating physician is determining if a presenting patient has true metastases versus the development of multiple primary tumors. Several criteria have been described for distinguishing multiple primary tumors lung cancer (MPLC) versus metastatic disease. The most widely cited of these are those outlined by Martini and Melamed [13] and recently summarized in a review by Pfannschmidt and Dienemann [14]. Typically, synchronous multiple primary lung cancer (SMPLC) was defined as those physically distinct and separate tumors were diagnosed within 6 months and histology was different, or when the tumors had similar histology and located in different lobes or lungs, in the absence of lymphatic metastases in the common drainage basins and extrathoracic metastases at the time of diagnosis. Metachronous multiple primary lung cancer (MMPLC) was defined as those tumors were diagnosed beyond 6 months and fulfilled the above criteria. For MPLC, aggressive local treatment such as stereotactic ablative radiotherapy was reported to achieve median survival of 46.5 months and overall survival of 67% at 3 years and 22.3% at 5 years [15]. The prognosis of OM is poorer than MPLC in lung cancer. In synchronous tumors, the following criteria indicate metastatic disease: (1) same segment, (2) no carcinoma in situ, or (3) carcinoma in lymph node drainage sites common to both lesions. For metachronous tumors, metastatic disease is defined by: (1) interval less than 2 years and in the same lobe, or (2) interval less than 2 years and lymph node drainage sites involved common to both lesions. Niibe et al. recently proposed that a concept dividing OM into two categories: one with controlled primary and another with uncontrolled primary [16]. In general, OM with controlled primary site, so-called oligorecurrence, has better prognosis than OM with uncontrolled primary [17]. This classification helps us to identify patients whose primary tumor has been controled by local therapy such as surgery or radiotherapy but develop OM that could benefit significantly with local therapy to the limited sites of OM. Selective patients in this group may be potentially curable with systemic therapy plus local ablative therapy or surgical resection. 

Of course, outside of the thorax, these criteria are not applicable. In most patients with a prior diagnosis of locoregionally confined NSCLC in which the primary tumor is treated and who subsequently develop a metastatic deposit of the same histology with no evidence of a separate primary tumor, it can be presumed that the disease is a metachronous metastatic recurrence. It has been shown that patients presenting with synchronous OM have poorer survival outcomes than those with metachronous OM, though as noted above, the optimal cutoff for distinguishing synchronous versus metachrounous OM has varied. For instance, Tanvetyanon performed a comprehensive review of patients that received adrenalectomy for OM NSCLC, 10 publications contributing 114 patients. Forty-two percent of patients had synchronous metastasis, defined as a disease-free interval (DFI) of ≤6 months. The authors found that overall survival (OS) was 12 months in those patients with synchronous metastasis, versus 31 months with metachronous OM [18]. In another study from Japan, investigators found that a DFI of at least 1 year was a prognostic factor for improved survival in patients with OM disease in the bone, lungs, and brain [19]. And in a study by Inoue et al. examining the role of stereotactic radiation to the brain and/or body in OM lesions, the authors found that the 5-year OS rate was 40% for patients with a DFI of ≥12 months and 10% for a DFI less than this period [20].

## 4. Prognostic Factors for Survival in Oligometastatic NSCLC 

### 4.1. Number of Sites

The number of sites that has been classified as OM disease has varied, as authors have defined patients with this entity as any burden from 1 to 5 sites of disease. Several studies have demonstrated, however, that patients who have a larger number of sites have poorer survival outcomes. In the general metastatic setting, investigators from the University of Chicago have shown that baseline whole body metabolic tumor burden, as indicated by F-18 fluorodeoxyglucose positron emission tomography (^18^F-FDG PET) scan, was associated with a poorer prognosis [24]. In the setting of OM disease treated with local therapy, Salama et al. reported their findings of stereotactic ablative body radiation (SABR) in patients with 1–5 sites of metastatic disease and a life expectancy of at least 3 months. The primary sites included lung, head and neck, breast, colon/rectum, and kidney. The authors found that patients with 1-2 lesions had significantly better survival outcomes than those with 3–5 metastatic lesions [25]. These results have been recently updated by the same institution examining only patients with NSCLC, and the authors found that greater than two sites of disease were associated with worse progression-free survival (PFS) [26]. A study by Rodrigues et al. assessing RT in the setting of oligometastatic brain metastasis found that the cumulative brain metastases volume was of borderline significance when examining intracranial control [8]. In general, it is reasonable to presume that, particularly in the setting of the pathophysiology of metastatic disease described above, the lower the number of OM sites, the better the clinical outcome. In addition to the number of OM sites, the organ involved may also have the impact in clinical outcome. In general, the involvement of liver or bone may carry worse prognosis compared with adrenal or brain although the published data is limited. 

### 4.2. Thoracic Disease Burden (T and N Stage)

Several studies have shown that patients with earlier T and N stages have better improved survival outcomes in OM disease. For instance, investigators from MD Anderson Cancer Center examined 84 patients with newly diagnosed NSCLC and a solitary brain metastasis. The authors found that with aggressive treatment to the primary site, patients with stage I disease had survival outcomes that were comparable to those without brain metastases, but survival outcomes were much lower in those patients with stage III disease and a solitary brain metastasis versus those with stage III disease alone [6]. In another analysis of 103 patients with metastatic brain metastases, including 98 with a single brain metastasis, Bonnette et al. found that patients with both lower T and N stage had improved survival rates, leading the authors to conclude that aggressive treatment to the primary site should be favored in those patients without mediastinal lymph node involvement [7]. And in a study assessing the role of metastastectomy in patients with stage IV NSCLC undergoing metastastectomy for extracranial and extraadrenal metastases, Salah et al. found that patients with stage III intrathoracic disease had 5-year survival rate of 0% versus 77% and 63% in those patients with stage II and I disease, respectively [27]. 

### 4.3. Histology

Similar to other stages of NSCLC, adenocarcinoma has been found to portend for a more favorable prognosis in OM disease. The study by Bonnette et al. described above found that patients with adenocarcinoma had improved survival outcomes compared to other histologic subtypes [7]. Iwasaki et al. attempted to elucidate prognostic criteria for patients with NSCLC and brain metastases in patients that underwent resection of either the lung or brain lesion. The authors found that an adenocarcinoma histology was estimated as a risk factor in their final model, along with node negative status and a normal carcinoembryonic antigen (CEA) level [9].

## 5. Data for Aggressive Local Therapy in Oligometastatic NSCLC by Site of Disease

### 5.1. Brain

Table 1 demonstrates selected studies of patients treated with local therapy in the setting of OM NSCLC [9–13]. Several points can be made from examining this table. First, the definition of oligometastatic varies among studies, from a solitary metastasis to up to 6 metastases. As a definition of 5 or less is consistent with most analyses in the literature, we would advocate these criteria in future analyses. Second, several of the prognostic factors above were shown to be correlated with survival outcomes, such as nodal status, histology, and synchronous versus metachronous disease. Finally, an aggressive approach to both the primary and the oligometastatic site was feasible and successful in selecting patients, and thus we would recommend considering a combined approach of systemic therapy with either resection or stereotactic radiosurgery (SRS) in patients with a solitary brain metastasis [17]. Patients with advanced nodal disease could be considered for such an approach, pending response to systemic treatment.

### 5.2. Adrenal Gland

There have been several small studies pertaining to aggressive treatment of the adrenal gland in the setting of OM NSCLC. As mentioned above, these studies have been pooled and analyzed by Tanvetyanon et al., who included 10 publications and 114 patients. The authors had the following findings: 42% of patients had synchronous metastases (DFI ≤ 6 months), with the remainder having metachronous lesions. Median DFIs were 0 and 12 months in these two groups, respectively. Second, serious complications from adrenalectomy in this setting were rare. Third, the 1- and 2-year OS rates were 80% and 52% for metachronous lesions and 45% and 30% for synchronous OM disease, while the 5-year survival rates were approximately 25% for each disease state [18]. A comprehensive review of prognostic factors in the setting of isolated adrenal metastases has not ever been performed to our knowledge, likely due to the small size of available studies. However, 5-year survival rates range from approximately 5 to >50% [18, 28–31], and we believe that similar prognostic factors can be extrapolated as has been observed in OM to the brain and mixed sites.

### 5.3. Studies Examining Aggressive Treatment to the Primary Site and Mixed Oligometastatic Sites

 Several studies have examined the impact of treating the primary site and all OM sites of disease regardless of location, as depicted in Table 2. Hanagiri et al. retrospectively investigated the outcomes of 36 patients who underwent surgical resection to the primary site for stage IV NSCLC between 1995 and 2008 for up to 5 sites of metastatic disease. The metastatic sites ranged from brain, adrenal gland, axillary lymph nodes, liver, and contralateral pulmonary metastases. The overall 5-year survival rate in this group of patients was 26.8%, with improved OS rates (though not statistically analyzed) in patients with negative lymph nodes at the time of treatment (28.3 versus 20.4%) [21]. And Guerra et al. recently analyzed the role of aggressive chemoradiation to the primary site in the thorax with or without treatment to the distant lesions in a variety of OM sites. The authors found that more aggressive thoracic radiation, as manifested by increased radiation dose, was associated with improved OS outcomes [22].

 One of the only prospective trials assessing the role of aggressive local therapy in the setting of OM disease was a phase II study performed at Memorial Sloan-Kettering Cancer Center. In this study, 23 patients with a synchronous solitary metastasis underwent three cycles of chemotherapy with mitomycin, vinblastine, and cisplatin (MVP) followed by resection of all disease sites and then two more cycles of VP therapy. The authors found that 12 patients completed induction chemotherapy, and 8 of these patients underwent R0 (microscopically negative margin) resections. Five patients had R0 resections without completing induction MVP. The median survival was 11 months, and 2 patients survived for 5 years without disease (<10%). The authors concluded that OS did not appear to be superior with this treatment strategy [23].

## 6. Treatment of Oligometastatic NSCLC: Where Are We Now?

Much has changed since the aforementioned prospective trial demonstrating no clear efficacy to an aggressive local approach after induction chemotherapy. First, over the past decade, radiation techniques have advanced greatly with the advent modalities such as intensity-modulated radiation therapy and stereotactic radiation. As a result, combined techniques of surgical resection and radiation can be used to more effectively treat residual sites of disease and minimize toxicity, both of which can be individualized based on the size and location of the disease, as well as a patient's anatomical characteristics. Second, targeted therapy has advanced systemic options, and patients can therefore be better selected for optimal treatment based on molecular characteristics. For example, randomized phase III trials have shown that patients with known epidermal growth factor receptor (EGFR) mutations experience prolonged survival outcomes compared with standard chemotherapy alone [32, 33]. Erlotinib is now Food and Drug Administration (FDA) approved for the treatment of first-line NSCLC patients bearing EGFR mutations. Similar advances are being made with anaplastic lymphoma kinase (ALK) inhibitors, which are effective in patients that have rearrangements of the ALK gene [34]. Finally, maintenance chemotherapy has been shown to provide survival benefits in patients with metastatic NSCLC, either in the continuation maintenance or switch maintenance setting. In terms of continuation maintenance, Eastern Cooperative Oncology Group (ECOG) 4599 demonstrated a benefit for bevacizumab [35] and the Paramount Phase III study showed an improvement in PFS for pemetrexed [36]. Similarly, in the switch maintenance setting, the SATURN study demonstrated an improvement in OS with erlotinib [37], while a similar improvement in survival was shown with pemetrexed in the JMEN study [38]. 

These advances create opportunities for the treatment of oligometastatic NSCLC. Utilizing the information gained from multiple retrospective studies, this question would ideally be answered with a prospective trial in which patients are randomized to novel systemic therapy followed by aggressive local therapy utilizing both surgery and modern radiation techniques. Maintenance therapy should also remain an option in this patient population when appropriate, and patients could be stratified or included/excluded based on the prognostic factors gleaned from the analyses above. Given the emerging biologic and clinical evidence that oligometastatic NSCLC is a separate disease entity when compared to widespread metastatic disease, ideally patients could receive selective aggressive local therapy based on their specific disease characteristics, similar to other oncologic scenarios in which personalized medicine is the ultimate goal. A phase II clinical study to address this issue is ongoing in MD Anderson Cancer Center.

## Figures and Tables

**Table 1:** Selected studies of local treatment in oligometastatic NSCLC with brain metastases.

Study	Year	*N*	Criteria	Treatment	Findings
Hu et al. [6]	2006	84	Solitary brain metastasis	SRS or surgery	Stage I intrathoracic patients had better OS outcomes than stage III
Bonnette et al. [7]	2001	108	Brain metastasis (98 with solitary)	Surgery	Adenocarcinoma, T stage, complete resection with better outcomes
Rodrigues et al. [8]	2011	66	≤6 intracranial lesions	Image-guided SIB RT	Presence of systemic disease, lower performance status correlated with decreased OS
Iwasaki et al. [9]	2004	41	Solitary brain metastasis	Resection of primary site and brain metastasis	Risk score criteria for improved OS: adenocarcinoma, node-negative, normal CEA level
Mussi et al. [10]	1996	52	Solitary brain metastasis	Resection of primary site and brain metastasis	No status, lobectomy associated with decreased OS. 5-year OS in patients with synchronous/metachronous lesions 6.6/19%, respectively
Machiarini et al.[11]	1991	37	Solitary brain metastasis. Synchronous (<1 month) and metachronous included.	Resection of primary site and brain metastasis	Most frequent site of first recurrence was ipsilateral thorax (*n* = 14) and brain (*n* = 6). The receipt of adjuvant chemotherapy was strongest predictor of disease-free interval
Wronski et al. [12]	1995	231	Single (87%) or multiple (13%) metastatic intracranial lesions	Resection	Female gender, complete location, infratentorial location, no systemic metastases, age < 60 years associated with improved OS

**Table 2:** Selected studies of local treatment in oligometastatic NSCLC with mixed metastatic sites.

Study	Year	N	Criteria	Treatment	Findings
Hanagiri et al. [21] (retrospective)	2011	36	Up to 5 metastastic sites, stage IV disease	Surgery or radiation	5-year OS with distant metastasis 30.1%, pleural dissemination 25.1%
Guerra et al. [22] (retrospective)	2012	78	Up to 5 synchronous metastatic sites, Definitive chemoRT (44 also underwent treatment to OM sites)	Surgery or radiation to OM sites	High radiation dose, performance status, lower intrathoracic tumor volume correlated with improved OS
Downey et al. [23] (prospective)	2002	23	Solitary synchronous lesions	MVP × 3, then surgery on all sites, then VP × 2	MVP poorly tolerated, 2/23 patients disease free at 5 years
